# The impact of fatigue on balance in trained long-distance runners

**DOI:** 10.1080/15502783.2025.2550175

**Published:** 2025-08-28

**Authors:** Kyle Woods, Jonathan Naylor

**Affiliations:** Walsh University, Department of Exercise Science, North Canton, OH, USA

**Keywords:** Running, balance, fatigue, post-exercise

## Abstract

**Background:**

Fatigue is extreme tiredness resulting from mental or physical exertion or illness and stands as an incredibly important indicator of running economy in highly trained athletes. It is well understood that fatigue has a negative effect on performance, but it is unclear how exactly fatigue impacts balance. The goal of this study was to determine the effect that fatigue has on balance after a period of relatively moderate exercise in a sample of trained college distance runners.

**Methods:**

Seventeen members of a division II collegiate cross-country team were studied (nine males, eight females) ages 18–23 years old. The participants first completed two pre-exercise balance tests, consisting of a Unilateral Stance Test (US) and an Adaptation Test (ADT) on the NeuroCom Balance Master. The US balance test included four conditions: right leg and eyes – open, left leg and eyes – open, right leg and eyes – closed, and left leg and eyes – closed. The ADT involved two conditions: toes-up and toes-down. Then, participants completed a moderate intensity endurance run of 45 minutes on a treadmill at a pace determined by the Karvonen method at 65–75% of the participant’s Heart Rate Reserve (HRR) at a 0% grade. Post-exercise, the balance tests were repeated. Paired samples t-tests were performed to determine if there was a significant effect of fatigue on any of the tested balance conditions.

**Results:**

The toes-up and toes-down conditions of the ADT suggested significant balance improvement post-exercise (sway scores of 70.62 + 11.20 v 57.37 + 11.17 *p* < .001, *t* = 6.735) (sway scores of 52.10 + 11.42 v 46.15 + 7.76 *p* < .014, *t* = 2.750). Combining the results of all trials into one score for the ADT again exhibited significant improvement in pre-post exercise balance (sway scores of 61.37 + 9.87 v 54.29 + 11.78 *p* < .043, *t* = 2.198). All other data were nonsignificant.

**Conclusion:**

The results of this study indicate that balance capabilities may improve after a bout of fatiguing exercise.

